# Effect of chitosan‐based coatings enriched with savory and/or tarragon essential oils on postharvest maintenance of kumquat (*Fortunella* sp.) fruit

**DOI:** 10.1002/fsn3.835

**Published:** 2018-11-08

**Authors:** Seyed F. Hosseini, Milad Amraie, Mohammad Salehi, Maedeh Mohseni, Hajer Aloui

**Affiliations:** ^1^ Faculty of Marine Sciences Department of Seafood Processing Tarbiat Modares University Noor Iran; ^2^ Department of Food Science and Industries Khazar Institute of Higher Education Mahmoodabad Iran; ^3^ Laboratoire des Substances Naturelles (LSN, LR10 INRAP02) Institut National de Recherche et d'Analyse Physico‐chimique (INRAP) Pôle Technologique de Sidi Thabet Sidi Thabet Tunisia

**Keywords:** chitosan coating, kumquat fruit, physicochemical properties, postharvest quality, sensory attributes

## Abstract

The present study assessed the ability of chitosan‐based coatings incorporating savory and/or tarragon essential oils (EOs) to preserve the postharvest quality of kumquats. Changes in weight loss, titratable acidity, total soluble solids, and vitamin C content were determined over 30 days of storage at 7°C. Savory (*Satureja hortensias* L.) essential oil was characterized by thymol (29.1%), carvacrol (26.6%), and γ‐terpinene (24.72%) as major constituents. While, in the tarragon (*Artemisia dracunculus* L.) essential oil, estragol (81.89%), β‐*cis*‐Ocimene (4.62%), and β‐*trans*‐Ocimene (3.44%) were the main ones. The CH‐EOs coatings were effective in reducing weight loss of kumquats fruits during storage. Moreover, the tested composite coatings showed positive effects in maintaining vitamin C and fruits treated with CH‐oil coatings retained good sensory acceptability. The obtained results demonstrate the potential of the combined application of chitosan and savory and/or tarragon EOs as a promising postharvest treatment for maintaining the postharvest quality of kumquats fruits.

## 1 INTRODUCTION

Kumquat (*Fortunella* sp.) is a smallest citrus fruit with a round‐oval shape that belongs to the Rutaceae family (Peng et al., 2013); it is native of China, but recently has been widely cultivated in the world. Fortunella genus is known for their beneficial effects in healing cough, cold, preventing blood vessels’ rupture, and blood capillaries’ permeability (Abirami, Nagarani, & Siddhuraju, 2014). Moreover, kumquats can be used as a potential source for the production of several products such as candied fruit, marmalades, wine, and sauces (Peng et al., 2013). A distinctive property that distinguishes kumquats from other citrus, is that they are eaten along with their peel, that is affluent in various chief essential oils (EOs) (containing limonene, caryophyllene, α‐humulene, α‐bergamotene, α‐muurolene, pinene), antioxidants, and fiber. Furthermore, kumquats are excellent source of vitamin C (73%), and has good quantities of B‐group vitamins (23.5%), as well as acceptable source of minerals (39%) (http://www.nutrition-and-you.com/kumquat-fruit.html). However, during storage the composition of citrus may be changed by alter of conditions such as temperature and storage period (Peng et al., 2013); changes in kumquats compounds are also correlated with the increased time of warehousing. Meanwhile, their character such as nutrition, favor, and appearance deteriorated during the process of storage and transportation owing to water loss, browning, decay, as well as mechanical injuries (Jianglian & Shaoying, 2013). To extend the shelf life of postharvest fruit, some effective methods including low temperature, modified atmosphere packaging, irradiation and coating, have been applied. Regarding this, application of edible films/coatings can be a cost‐effective approach to prevent physical injury, enhance appearance, and reduce fruits decay (Guerra et al., 2016). Edible coatings are thin layer of biodegradable material (polysaccharide, protein, and lipid) which form directly on the surface of fresh fruit and protect their quality by transaction of oxygen and moisture, prevent of color and flavor loss, as well as reduce of fresh fruit respiration (Guerra et al., 2015). Chitosan (CH), a cationic, biodegradable, and nontoxic hydrocolloid that obtained by deacetylation of chitin, is a famous material that uses for production of antimicrobial edible‐based coatings (Bonilla, Poloni, & Sobral, 2018; Hosseini & Gómez‐Guillén, 2018). The beneficial effects of chitosan edible coating were seen in terms of extended storage life, quality maintenance, and postharvest pathogen control. Studies have shown that CH‐based edible coatings are able to maintain the quality and extending the shelf life of some fruits such as plum (Kumar, Sethi, Sharma, Srivastav, & Varghese, 2017), blueberries (Mannozzi et al., 2018), and guava (Nair, Saxena, & Kaur, 2018).

In the last few years, considerable attention has been directed toward natural compounds, such as essential oils (EOs) as an efficient tool for maintaining fruits postharvest quality (Aloui et al., 2014). EOs are volatile and bioactive materials which have various properties like antioxidant and antibacterial (Hasani, Ojagh, & Ghorbani, 2018; Hosseini, Rezaei, Zandi, & Farahmandghavi, 2015). Because CH and EOs are generally recognized as safe (GRAS) by the FDA, their application in the formulation of coatings as fruits keeping approaches has been simplified (Guerra et al., 2016).

The genus *Satureja* consist of over 30 species which belonging to Lamiaceae family that are mainly distributed in the Mediterranean region (Momtaz & Abdollahi, 2010); *Satureja hortensis* L. (summer savory) is a well‐known *Satureja* species, which grows in Iran. Iranian people used the aerial parts of this species for treat of various ailment for instance gastroenteritis, diarrhea, and wound infections (Behravan, Ramezani, Kasaian, & Sabeti, 2004). Furthermore, EOs of *Satureja* species are known for their antioxidant and antimicrobial activities which are attributed to their main volatile compounds such as thymol (44.5%) and γ‐Terpinene (23.9%) (Sefidkon & Jamzad, 2000). Besides *Satureja* genus, *Artemisia* genus contain of considerable number of species which *Artemisia dracunculus* L. (tarragon) is one of the species; this plant grows in different world areas and belonging to the Asteraceae family. EOs isolated from *A. dracunculus* showed an antitumor, antifungal, and DNA anti‐damaging properties (Meepagala, Sturtz, & Wedge, 2002); also EOs of this genus has been used as sedative and anticonvulsant. To our knowledge, no data is accessible about the incorporation of the essential oils of savory (SEO) or tarragon (TEO) in chitosan‐based coatings. To the best of our knowledge, the application of CH coating in combination with savory or tarragon essential oils for postharvest treatment of kumquats has not been studied to date. Therefore, the purpose of the present study was to assess the effect of the combined application of CH and SEO or TEO as a postharvest treatment on the physicochemical and sensory characteristics of fresh kumquat fruit throughout 30 storage days at 7°C.

## 2 MATERIALS AND METHODS

### 2.1 Materials

Kumquat fruit (*Fortunella* sp.) (identical in size and shape, bright orange in color with a healthy appearance) was kindly provided from a fruit farming located in Mahmoudabad (Mazandaran, Iran). CH (medium molecular weight, 75%–85% degree of deacetylation) was supplied by Sigma‐Aldrich (St. Louis, MO, USA). Sodium hydroxide (NaOH), acetic acid, and other chemicals were purchased from Merck Chemicals Co. (Darmstadt, Germany). Savory and Tarragon EOs were obtained from Barij Essence Pharmaceutical Co. (Kashan, Iran) and stored in dark container at 4°C until used.

### 2.2 EOs analysis and identification procedure

The individual constituents of EOs were identified via GC/MS by a Thermoquest‐Finnigan instrument (Thermo Fisher Scientific, USA) Model 2000R supplied with a capillary DB‐5 fused silica column (30 m × 0.25 mm *i.d*., film thickness 0.25 μm). The oven temperature was set up to increase from 50 to 250°C at a rate of 2.5°C/min and finally held for 30 min; transfer line temperature was 250°C. As the carrier gas, helium was applied at a flow rate of 1.5 ml/min with a split ratio equal to 1/50. Mass spectra were captured at 70 eV.

### 2.3 Preparation of CH coatings containing EOs

Coating solutions were prepared by dissolving CH powder (4 mg/ml) in acetic acid (1 ml/100 ml) under agitation (120 rpm) during 6 hr. Subsequently, EO of savory (SEO), tarragon (TEO), or combination of both EOs (SEO+TEO) were added at concentration of 1.25 or 2.5 μl/ml (Guerra et al., 2016), followed by stirring for an additional 18 hr at room temperature; then, glycerol was added (2 ml/100 ml) as a plasticizer.

### 2.4 Application of the coatings

Whole kumquats were dipped in a solution of sodium hypochlorite (1%, v/v) for 15 min, washed (to remove chlorine traces), and then dried for 2 hr in a safety cabinet. Selected kumquats were randomly distributed into four groups. A control (uncoated) group was immersed in sterile distilled water containing glycerol, and the other three‐ones were treated with each one of the coatings (CH‐SEO, CH‐TEO, and CH‐S+TEO). Kumquats samples were immersed in the corresponding coating solutions for 3 min, allowed to dry at room temperature for 1 hr and, afterward, cold stored on PET containers in refrigerator at 7 ± 1°C. A total of 45 fruits for each time/treatment were characterized as to the different properties described below, at different cold storage times (0, 10, 20, and 30 days) (Guerra et al., 2015).

### 2.5 Physicochemical properties evaluation

#### 2.5.1 Weight loss determination

The weight of the fruit was monitored at different storage time intervals (0, 10, 20, and 30 days), and the percentage of weight loss was calculated by the following equation:

$$\text{weight}\;\text{loss}=\frac{w_{i}-w_{t}}{w_{i}}\times 100$$

(w_i_): the initial weight of the fruit before maintenance

(w_t_): weight of the sample after maintenance till t time

#### 2.5.2 Determination of titratable acidity (TA) and total soluble solids (TSS)

The TA was determined using phenolphthalein as an indicator with 0.1 N NaOH, and the results were demonstrated as mmol H^+^/100 g of fruit (equivalent of citric acid); the TA was calculated from Equations (2). The TSS content was ascertained by a hand refractometer (Atago, PaL‐1, Tokyo, Japan), and the results were expressed as Brix (Guerra et al., 2015).

$$\text{TA}=\frac{V\times N\times 0.064}{C}$$

(V): exhausted sodium hydroxide volume

(N): exhausted sodium hydroxide normality

(C): Volume of titrated juice

### 2.6 Vitamin C content

The vitamin C content in kumquat was determined by titration method with 2,6‐dichlorophenol indophenol (DIP). In this way, 5 ml of metaphosphoric acid (3%, v/v) and 2 ml of distilled water added to 10 ml of juice sample and the mixture was titrated with DIP till appearing purple color. After putting in the equation, the amount of vitamin C can be calculated and expressed as mg/100 g sample (Lee & Coates, 1999):

$$\text{Vit}\;C\left(\text{mg}/100g\right)=\frac{A}{V_{2}-V_{1}}\times B$$

(A): exhausted volume of DIP for 1 ml of juice

(V_1_): exhausted volume of DIP for 1 ml blank soluble

(V_2_): exhausted volume of DIP for 1 ml pure vitamin C

(B): volume of juice in 100 g of sample

### 2.7 Sensory evaluation

Kumquat coated with CH‐based coatings containing SEO, TEO, or S+TEO were subjected to sensory tests at different time intervals (0, 10, 20, and 30 days). The analysis was performed under controlled conditions of light and temperature in individual booths. Each panelist received four kumquats treated with the different CH‐EOs coatings served on disposable white plates coded with a random four‐digit number. The tasters were asked to eat a cracker and drink water between samples to avoid aftertaste effects. For the acceptability of appearance, aroma, flavor, texture, and overall assessment, a seven‐point structured hedonic scale was used, which ranged from one (dislike very much) to seven (like very much) (Guerreiro, Gago, Faleiro, Miguel, & Antunes, 2015).

### 2.8 Statistical analysis

Analysis was done with the SPSS 19.0 software (Chicago, USA). Multi‐factor ANOVA and Duncan's multiple‐range test with a 95% significance level was performed for comparisons among treatments.

## 3 RESULTS AND DISCUSSION

### 3.1 Essential oils composition

GC‐MS analysis of SEO and TEO resulted in the identification of 32 and 33 compounds, respectively (Table 1). The major constituents in SEO were thymol (29.1%), followed by carvacrol (26.6%), γ‐terpinene (24.72%), ρ‐cymene (7.55%), and α‐terpinene (3.96%). Estragol (81.89%) was the major compound in TEO, followed by β‐*cis*‐Ocimene (4.62%), β‐*trans*‐Ocimene (3.44%). The other identified constituents in SEO and TEO ranged from 0.1%–1.68% and 0.05%–1.67%, respectively. Various compounds found in these EOs, namely carvacrol, thymol, and γ‐terpinene, have acknowledged antioxidant attributes (Teixeira et al., 2013). Additionally, antibacterial properties have also been described for carvacrol, thymol, ρ‐cymene, α‐terpinene (Javidi, Hosseini, & Rezaei, 2016).

**Table 1:** GC‐MS analysis of the essential oils from savory (SEO) and tarragon (TEO)

SEO			TEO		
Compound	RI^a^	%	Compound	RI^a^	%
α‐Thujene	929	1.24	1‐R‐α‐Pinene	932	0.64
α‐Pinene	937	0.71	Camphene	943	0.05
Camphene	950	tr^b^	Sabinene	968	0.05
Sabinene	991	tr	β‐Pinene	974	0.09
β‐Pinene	984	0.35	β‐Myrcene	986	0.11
β‐Myrcene	993	1.68	L‐Limonene	1,027	1.67
α‐Phellandrene	1,040	0.33	β‐*trans*‐Ocimene	1,039	3.44
d‐3‐Carene	1,011	tr	β‐*cis*‐Ocimene	1,050	4.62
α‐Terpinene	1,021	3.96	α‐Terpinolen	1,085	0.33
β‐Phellandrene	1,032	0.55	Allo‐Ocimene	1,128	0.71
1,8‐Cineole	1,037	tr	Estragol	1,215	81.89
γ–Terpinene	1,059	24.72	Pulegone	1,240	0.05
α–Terpinolene	1,146	tr	Bornyl acetate	1,287	0.19
*cis*‐sabinene hydrate	1,067	tr	Thymol	1,290	0.14
Linalool	1,104	tr	Carvacrol	1,306	0.31
Borneol	1,164	tr	δ‐Elemene	1,335	0.09
ρ‐Cymene	1,185	7.55	Eugenol	1,358	0.44
α‐Terpineol	1,190	tr	(E)‐Methyl cinnamate	1,380	0.43
*cis*‐Dihydro carvone	1,195	tr	Eugenol methyl ether	1,404	1.49
Carvacrol methyl ether	1,245	0.1	Caryophyllene	1,418	0.1
Thymol	1,290	29.1	Decalactone	1,467	0.17
Carvacrol	1,299	26.6	Germacrene D	1,479	0.21
Thymol acetate	1,352	0.3	β‐Ionone	1,482	0.09
Carvacrol acetate	1,371	0.1	Bicyclogermacrene	1,494	0.16
T‐Caryophyllene	1,415	0.52	α‐Farnesene	1,504	0.1
Aromadendrene	1,432	tr	β‐Sesquiphellandrene	1,521	0.09
Neryl acetate	1,436	tr	(−)‐Spathulenol	1,578	0.17
α‐Humulene	1,452	tr	Caryophyllene oxide	1,583	0.14
β‐Ionone	1,483	tr	Spathulenol	1,609	0.91
β‐Bisabolene	1,508	0.99	Caryophyllenyl alcohol	1,647	0.05
Spathulenol	1,574	0.12	Herniarin	1,717	0.13
α‐Bisabolol	1,681	tr	2‐Pentadecanone,6,10,14‐trimethyl‐	0.06	0.06
			1,2,4‐Triazolo[4,3‐a]pyridine,3‐phenyl‐	0.2	0.2

a Retention indices, using paraffin (C5–C25) as references.

b tr, traces (<0.05%).

### 3.2 Physicochemical properties

#### 3.2.1 Weight loss

**Figure 1:**
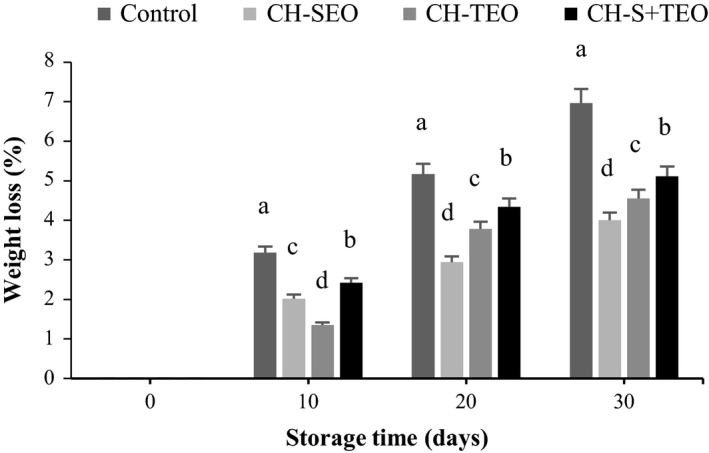
Changes in weight loss of uncoated and coated kumquats during storage. Different letters indicate statistically significant differences (*p *<* *0.05)

Weight loss in fresh fruit is principally owned by the water loss caused by transpiration and respiration; the loss of carbon during respiration can also be caused by some weight loss. Figure 1 shows the changes in weight loss both for uncoated and coated samples throughout the storage time. As expected, weight loss increased during storage in all samples, and was more intensified for the control samples (uncoated) than for the CH‐EOs coated ones, presumably revealing the improved structural continuity of the used coatings as well as EOs hydrophobic character as efficacious obstacle versus gases, moisture, and solute movement (Guerra et al., 2015). It is known that migration of water from fruit and the loss of carbon atoms from fruit in each cycle of respiration is the major cause of fruit weight loss during storage; CH coating inhibits the water vaporization by creation of layer on the surface and decrease metabolic processes and respiration (Guerra et al., 2016). Our results are supported by Ali, Muhammad, Sijam, and Siddiqui (2011), where water loss of papaya fruit can be reduced by coating with CH. Furthermore, EOs have hydrophobic nature and its incorporation into CH‐based coatings can control and decrease the rate of mentioned processes (Sánchez‐González et al., 2011). Guerra et al. (2015) demonstrated that the cherry tomato fruits coated with CH‐*Mentha piperita* L. EO (MPEO) or CH‐*Mentha* × *villosa* Huds essential oil (MVEO) exhibited comparatively lower weight loss, implying the enhanced structural continuity of the applied coatings and hydrophobic nature of the incorporated EOs as effective barriers against gases, moisture, and solute movement. However, as shown in Figure 1, the rate of weight loss was higher (*p *<* *0.05) in CH‐S+TEO coated fruits compared with individual CH‐EOs treatments throughout the assessed storage period. This is probably due to the competition between both essential oils (i.e., SEO and TEO) compounds in bonding to the CH and/or antagonistic effect of essential oils (Goñi et al., 2009). On the last day of storage, the uncoated samples underwent an escalation of weight loss, which can be assigned to raise in the metabolic activity of fruits, due to tissue senescence over long storage times (Sánchez‐González et al., 2011).

#### 3.2.2 Titratable acidity (TA) and total soluble solid (TSS)

For determination of maturity and sour taste in citrus fruits, titratable acidity is a key parameter; fruit maturity is one of the most important factors to decide how well fruit will store (http://hannainst.com/hi84532-titratable-acidity-mini-titrator-for-fruit-juice-analysis.html).

**Figure 2:**
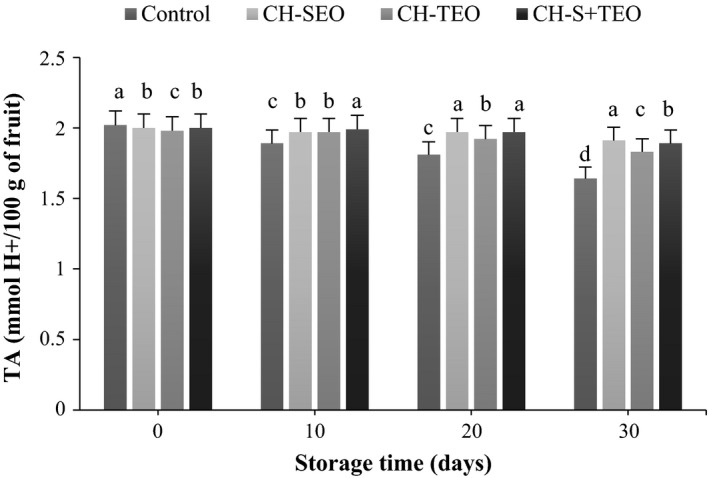
Changes in TA of uncoated and coated kumquats during storage. Different letters indicate statistically significant differences (*p *<* *0.05). TA, titratable acidity

In general, TA values in all samples were reduced overtime (Figure 2), which can be attributed to the increase in ethylene production and respiration rate during the advent of ripening (Oz & Ulukanli, 2012). However, the decrease of TA values was affected by different type of CH/EOs treatments, as CH‐SEO coating treatment had higher TA than the other ones. In this research, retention of the TA content could be attributed to the CH‐based coating, which could control the permeability of CO_2_/O_2_ and slowed down ethylene production and ripening rate of fruit (Dong, Cheng, Tan, Zheng, & Jiang, 2004). Hong, Xie, Zhang, Sun, and Gong (2012) reported that the chitosan coating slowed down the changes in TA of guava fruit, effectively delaying fruit ripening. Retaining of titratable acidity was also reported by Das, Dutta, and Mahanta (2013) for tomatoes coated with rice starch‐based edible coating containing coconut oil and tea leaf extract.

**Figure 3:**
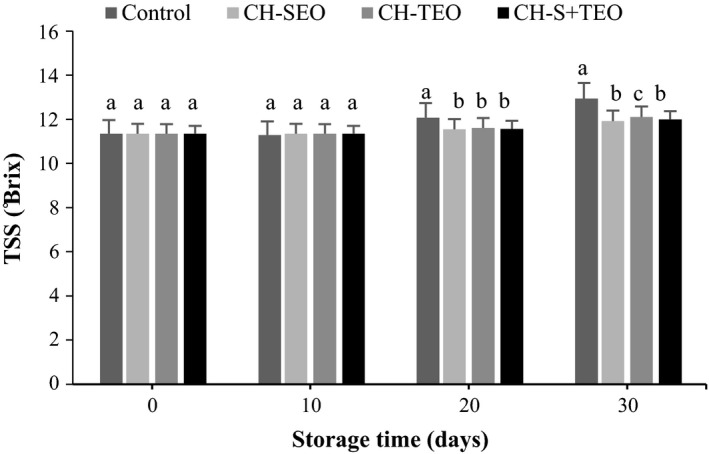
Changes in TSS of uncoated and coated kumquats during storage. Different letters indicate statistically significant differences (*p *<* *0.05). TSS, total soluble solids

Total soluble solids content increased throughout the storage in agreement with the progress of the ripening process, in which the highest soluble solids content being those of control samples, followed by those treated with CH‐TEO, CH‐T+SEO, and CH‐SEO coatings (Figure 3). Also, an increase in Brix value in this study may be correlated with the weight loss due to the humidity decrease in fruit, which can increase the concentration of TSS (Thompson, 2015). Remarkable differences (*p *<* *0.05) between uncoated (control) samples and the CH‐EOs coated ones were found on day 20 and 30 of storage. The effect of CH coating on TSS of kumquat fruits was probably due to the slowing down of respiration and metabolic activity, hence retarding the ripening process. Our results are in‐line with those Ali et al. (2011) and Hong et al. (2012), where a slow rise in TSS was recorded in papaya and guava fruits treated with CH.

### 3.3 Vitamin C content

**Figure 4:**
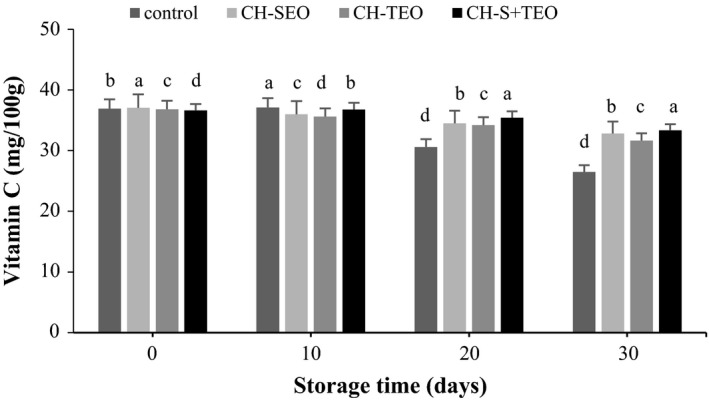
Vitamin C content of uncoated and coated kumquats during storage. Different letters indicate statistically significant differences (*p *<* *0.05)

Figure 4 shows changes in the total vitamin C contents of coated and uncoated kumquats during 30 days storage. The initial vitamin C content of kumquat fruit was 37.09 mg/100 g. Vitamin C retention was affected by the CH‐EOs coating as well as storage time. Even though vitamin C of both coated and uncoated samples decreased throughout storage, the application of CH‐based edible coatings markedly lowered the loss of vitamin C in kumquats. After 30 days of storage, vitamin C retention of fruits treated with CH‐SEO, CH‐TEO, and CH‐S+TEO coatings was 32.80, 31.65, and 33.33 mg/100 g, respectively, whereas control samples maintained 26.5 mg/100 g of initial vitamin C content. Present study showed that vitamin C content of CH‐S+TEO‐coated kumquats was highest among all the treatments at the end of storage. Since vitamin C loss can be markedly influenced by the existence of O_2_, the application of CH‐based coating formulations may reduce O_2_ diffusion, slow down the ripening rate and thereupon better preserve vitamin C contents and delay senescence of fruits (Xing et al., 2011). Similar results have been reported that apricots and green peppers kept O_2_ away from it, the loss of their vitamin C was delayed (Ayranci & Tunc, 2004). Furthermore, CH‐EOs coating could inhibit vitamin C loss, due to the protection effected by antioxidant phenolics in the oil (Xing et al., 2011).

### 3.4 Sensory quality

Changes in sensory attributes including appearance, tissue strength, aroma, flavor, and general acceptance of uncoated and coated kumquats during storage are tabulated in Table 2. While, up to 10 days of storage, no difference was observed (*p *>* *0.05) in the appearance values between control and fruits treated with CH‐based coatings containing different EOs (savory, tarragon, savory‐tarragon), the treated samples presented scores higher than that of uncoated fruits at the end of storage period (Table 2). At day 30, panelist observed white parts on control kumquat fruits, which became unacceptable for consumption; whereas the quality of the CH‐oil coated kumquats was retained, because of the characteristics of high transparency and luminosity presented by chitosan‐based coatings and or/films (Guerra et al., 2016).

**Table 2:** Mean values of sensory attributes in uncoated and coated kumquats during storage

Attributes	Treatments	Days of storage			
		0	10	20	30
Appearance	Control	6.0 ± 1.0^Aa^	6.0 ± 0.5^Aa^	5.2 ± 0.2^Ac^	3.2 ± 0.5^Bb^
	CH‐SEO	6.0 ± 1.0^Aa^	6.0 ± 0.5^Aa^	5.8 ± 0.05^Aa^	5.6 ± 0.3^Aa^
	CH‐TEO	6.0 ± 1.0^Aa^	6.0 ± 0.5^Aa^	5.6 ± 0.15^Aab^	5.5 ± 0.7^Aa^
	CH‐S+TEO	6.0 ± 1.0^Aa^	6.0 ± 0.5^Aa^	5.4 ± 0.07^Abc^	5.3 ± 0.5^Aa^
Tissue strength	Control	6.5 ± 0.5^Aa^	5.5 ± 0.5^Ab^	4.0 ± 0.5^Bb^	2.0 ± 1.0^Cb^
	CH‐SEO	6.5 ± 0.5^Aa^	6.3 ± 0.2^Aa^	5.9 ± 0.4^Aba^	5.5 ± 0.1^Ba^
	CH‐TEO	6.5 ± 0.5^Aa^	6.3 ± 0.3^Aba^	5.7 ± 0.2^Ba^	5.1 ± 0.1^Ca^
	CH‐S+TEO	6.5 ± 0.5^Aa^	5.8 ± 0.2^Bab^	5.5 ± 0.4^Ba^	4.7 ± 0.3^Ca^
Aroma	Control	6.5 ± 0.5^Aa^	6.3 ± 0.2^Aa^	5.6 ± 0.4^Aa^	3.8 ± 0.2^Ba^
	CH‐SEO	6.0 ± 0.5^Aa^	5.8 ± 0.4^Aab^	5.5 ± 0.1^Aa^	4.5 ± 0.5^Ba^
	CH‐TEO	6.0 ± 0.5^Aa^	5.8 ± 0.3^Aab^	5.5 ± 0.2^Aa^	4.5 ± 0.5^Ba^
	CH‐S+TEO	5.8 ± 0.3^Aa^	5.5 ± 0.5^Ab^	5.3 ± 0.1^Aa^	4.5 ± 0.4^Ba^
Flavor	Control	6.0 ± 0.5^Aa^	5.7 ± 0.3^Aa^	4.9 ± 0.1^Ba^	3.5 ± 0.1^Cb^
	CH‐SEO	5.5 ± 0.5^Aa^	5.1 ± 0.2^ABb^	5.0 ± 0.7^Aba^	4.5 ± 0.3^Ba^
	CH‐TEO	5.5 ± 0.5^Aa^	5.2 ± 0.4^ABab^	5.0 ± 0.5^Aba^	4.3 ± 0.4^Ba^
	CH‐S+TEO	5.5 ± 0.5^Aa^	5.5 ± 0.2^Aab^	5.2 ± 0.3^ABa^	4.8 ± 0.2^Ba^
General acceptance	Control	6.0 ± 0.5^Aa^	5.2 ± 0.8^ABa^	4.5 ± 0.15^Bc^	3.0 ± 0.5^Cb^
	CH‐SEO	5.5 ± 0.5^Aa^	5.6 ± 0.4^Aa^	5.1 ± 0.15^Ab^	4.3 ± 0.2^Ba^
	CH‐TEO	5.5 ± 0.5^Aa^	5.5 ± 0.5^Aa^	5.1 ± 0.1^ABb^	4.1 ± 0.1^Ba^
	CH‐S+TEO	5.5 ± 1.0^ABa^	5.8 ± 0.35^Aa^	5.4 ± 0.1^Aa^	4.4 ± 0.4^Ba^

*Notes*

^A–C^For each trial, different superscript letters in the same row denote differences (*p *<* *0.05) among the mean values (for the same treatment at different storage periods) according to Duncan's multiple‐range test.

^a–c^For each trial, different subscript letters in the same column denote differences (*p *<* *0.05) among the mean values (for the different treatments at a same storage period) according to Duncan's multiple‐range test.

The sensory test displayed a significantly different (*p *<* *0.05) perception of the panelists concerning the tissue strength of the samples treated with CH‐EOs coatings as compared to uncoated samples (Table 2). CH coating incorporated with EOs delayed ripening and retained greater firmness by protective effect on the cell wall of the coated fruits (Hernández‐Muñoz, Almenar, Del Valle, Velez, & Gavara, 2008); it is well known that hemicellulose and lignin as cell wall components have essential role in stability of fruit texture. In Table 2, it is visible that up to 10 day storage, the uncoated kumquats had a higher flavor scores in the sensory panel scale, while after this time, CH‐EOs coating delayed the flavor loss and resulted in higher scores, in comparison with uncoated fruits; in a lot of fresh fruits, loss of taste quality often caused by premature senescence.

Aroma is an important attribute of ripe fruit, which has great effect on their attractive; aroma refers to the smell of a fruit or vegetable product that generated by the production of various volatile compounds (Barrett, Beaulieu, & Shewfelt, 2010). The application of the CH‐based coatings containing EOs did not markedly (*p *<* *0.05) change the aroma characteristic of kumquats during storage (Table 2). This finding is worthy of note because of the possible negative effects of EOs on the sensory characteristics of food, particularly its food odor, which is usually explained as a restraining factor for the realistic application of EOs as antimicrobials in food keeping systems (Guerra et al., 2016). Total acceptability decreased throughout the storage for all samples (Table 2), especially uncoated fruits, but treated ones obtained higher score at the end of storage period; among them, kumquats coated with CH‐TEO treatment had the lowest score than other coated ones.

## 4 CONCLUSIONS

Edible coatings obtained from chitosan with savory (SEO) and/or tarragon (TEO) essential oils were proven to be effective at reducing weight loss and maintaining titratable acidity and vitamin C content. Moreover, Samples treated with CH‐EOs‐based coatings retained also good sensory acceptability compared with the uncoated ones which became unacceptable at the end of the storage period. These findings reveal the potential of the combined application of chitosan and SEO or TEO as an active packaging system for maintaining postharvest quality of kumquat fruits.

## CONFLICT OF INTEREST

The authors declare that they do not have any conflict of interest.

## ETHICAL STATEMENT

This study does not involve any human or animal testing.
